# Development of Grape Seed Extract-Fortified Biscuits: Effects on Dough Rheology, Biscuit Quality, Antioxidant Activity, and Sensory Evaluation

**DOI:** 10.3390/foods15162944

**Published:** 2026-08-21

**Authors:** Renrui Jiao, Fujuan Zhang, Li Yang, Cuntang Wang

**Affiliations:** 1 College of Food and Bioengineering, Qiqihar University, Qiqihar 161006, China; 2 Engineering Research Center of Plant Food Processing Technology, Ministry of Education, Qiqihar 161006, China; 3 College of Biochemical and Food Engineering, Chaoyang Normal University, Chaoyang 122000, China

**Keywords:** grape seed extract, low-gluten flour, biscuit, antioxidant activity

## Abstract

Heightened consumer awareness of health benefits has propelled nutritionally enhanced cereal products to the forefront of food industry innovation. The present work focused on manufacturing polyphenol-enriched biscuits by incorporating grape seed extract (GSE). The study evaluated different formulations of wheat flour with GSE at addition ratios of 0%, 0.2%, 0.4%, 0.6%, 0.8%, and 1% (*w*/*w*). Within the 0.2–0.6% GSE range, significant improvements were observed in dough properties and biscuit quality. Specifically, the dough development time and degree of softening decreased, while the dough stability time, peak viscosity, storage modulus, and loss modulus increased. The fortified biscuits had a darker color, lower baking loss rate, and increased spread factor, springiness, and cohesiveness. In addition, total phenolic content (TPC) and total flavonoid content (TFC) of the fortified biscuits increased significantly (*p* < 0.05), and the antioxidant activity of the biscuits was also enhanced. Sensory evaluation results indicated that biscuits with 0.6% GSE addition achieved the highest overall acceptability score (8.7 ± 0.4 on a 9-point hedonic scale), indicating good consumer acceptance. These findings identify 0.6% GSE as an appropriate fortification level for improving biscuit quality while enhancing polyphenol content and antioxidant capacity, providing a practical basis for the development of polyphenol-enriched bakery products.

## 1. Introduction

The increasing demand for healthier foods has accelerated the development of bakery products enriched with bioactive compounds, particularly polyphenols [1]. Biscuits are widely consumed due to their convenience and sensory appeal; however, those produced mainly from refined flour, fat, and sugar generally provide limited nutritional benefits and contain relatively low levels of natural antioxidants [2,3]. Moreover, oxidative deterioration during storage can negatively affect flavor, texture, and overall product quality. Therefore, improving the antioxidant potential of biscuits while maintaining desirable sensory properties remains an important challenge in bakery product development. Plant-derived extracts rich in phenolic compounds have attracted considerable attention as functional ingredients because of their antioxidant and bioactive properties [4,5]. Previous studies have demonstrated that polyphenol-rich ingredients, such as pomegranate peel extract, coffee silverskin, and apple pomace, can enhance phenolic content and antioxidant activity while modifying the physicochemical properties of biscuits and dough systems [6,7,8].

Grapes are among the most widely cultivated fruits worldwide, and their processing generates large amounts of seeds, skins, and pomace [9]. These grape-derived materials differ substantially in composition and technological functionality. Grape pomace is a heterogeneous mixture mainly consisting of grape skins and seeds. Grape seed powder contains insoluble dietary fiber, lipids, proteins, and phenolic compounds, whereas grape skin extracts are characterized by skin-derived phenolic compounds, including anthocyanins in colored grape varieties [10,11]. In contrast, grape seed extract (GSE) is a concentrated grape-derived ingredient that is particularly rich in proanthocyanidins and flavan-3-ols, including catechin and epicatechin, together with phenolic acids such as gallic acid [12]. Therefore, the effects of grape-derived powders cannot be directly extrapolated to GSE, as insoluble components and phenolic concentrations differ considerably among these materials. GSE has been recognized as a safe food ingredient and has shown potential for improving the nutritional and functional properties of cereal-based products [13,14]. Previous studies have reported that GSE-related ingredients improved antioxidant capacity and textural properties in biscuits, noodles, breads, and waffles [15,16,17]. However, the specific effects of concentrated GSE on dough behavior and biscuit quality require further investigation.

Although GSE has been increasingly investigated in food applications, its concentration-dependent effects in low-gluten biscuit systems remain insufficiently understood. Previous studies have mainly focused on bread and noodle products or on grape-derived ingredients such as grape seed powder and grape pomace, whereas comprehensive evaluations integrating dough processing properties, rheological behavior, biscuit quality, antioxidant activity, and sensory acceptance are limited. Moreover, the appropriate GSE supplementation level that balances technological performance, functional enhancement, and consumer acceptability has not been well established. Therefore, this study aimed to systematically investigate the effects of different GSE supplementation levels on the physicochemical and rheological properties of low-gluten flour dough, as well as the quality, antioxidant activity, and sensory characteristics of biscuits. The findings provide insights into the application of GSE as a polyphenol-rich ingredient for improving the technological and antioxidant-related properties of bakery products.

## 2. Materials and Methods

### 2.1. Materials

Grape seed extract (GSE) was purchased from Shandong Jinsheng Biotechnology Co., Ltd. (Linyi, China). According to the manufacturer’s specifications, the extract was prepared from grape pomace generated during wine and juice processing and contained ≥95% total polyphenols, ≥90% proanthocyanidins, and ≥70% oligomeric proanthocyanidins (OPCs). GSE was stored in a sealed, light-protected container at room temperature until use. Low-gluten wheat flour was purchased from Yihai Kerry Arawana Holdings Co., Ltd. (Harbin, Heilongjiang, China). The flour contained 9.5% protein, ≤14.5% moisture, and 75.0 g/100 g of carbohydrates, with a wet gluten content of ≥26%. Butter, sucrose, and fresh eggs (60 ± 3 g per egg) were obtained from local supermarkets in Qiqihar, China.

### 2.2. Determination of Farinograph Properties of Low-Gluten Flour

Farinograph characteristics were determined using a farinograph (JFZD 300, Beijing Dongfu Jiuheng Instrument Technology Co., Ltd., Beijing, China) according to Bai-Ngew et al. [18]. Briefly, 300 g of flour (14% moisture basis) was mixed with GSE at 0%, 0.2%, 0.4%, 0.6%, 0.8%, or 1% (*w*/*w*, flour basis) at 30 °C. Following 1 min pre-mixing, distilled water was automatically added to reach 500 BU consistency, and the dough was mixed at 60 r/min for 20 min with a plastic covering to prevent evaporation. Parameters, including water absorption (WA), dough development time (DT), stability (ST), degree of softening (DS), and farinograph quality number (FQN), were recorded. Measurements were performed using six independent samples.

### 2.3. Determination of Gelatinization Properties of Low-Gluten Flour

The gelatinization characteristics of wheat flour were measured using a Rapid Visco Analyzer (RVA 4500, Shanghai Ruibin International Trading Co., Ltd., Shanghai, China) according to the method of Zhu et al. [19] with slight modifications. Wheat flour (3.0 g, 14% moisture basis) was dispersed in 25 mL of distilled water in an aluminum RVA canister. The slurry was manually homogenized using a plastic paddle to prevent lump formation. The temperature profile was programmed as follows: holding at 50 °C for 1 min, heating from 50 °C to 95 °C at a rate of 12 °C/min, maintaining at 95 °C for 2.5 min, cooling from 95 °C to 50 °C at a rate of 12 °C/min, and holding at 50 °C for 2 min. The stirring speed was set at 960 rpm for the initial 10 s, followed by 160 rpm for the remainder of the test. The gelatinization temperature, peak viscosity, breakdown, and setback value were analyzed using the instrument support software.

### 2.4. Determination of the Dynamic Rheological Properties of Low-Gluten Flour

A rotational rheometer (Kinexus Pro+, Netzsch-Gerätebau GmbH, Selb, Germany) was employed for dynamic rheological analysis of the dough, according to the procedure reported by Zhou et al. [20], subject to minor adjustments. Firstly, the dough was prepared by blending the raw materials for 5 min with a farinograph. Parallel-plate geometry (diameter: 25 mm; gap: 1.0 mm) was used for all measurements. After the dough was prepared, paraffin wax was evenly applied to the edges of the testing plates to avoid moisture loss. After equilibrating at 25 °C for 30 min, the dough underwent strain sweep analysis (25 °C, 1 Hz, strain range: 0.01–100%) to establish the linear viscoelastic range (LVR). The LVR was determined to be within 0.1–5% strain, and a fixed strain of 1% was subsequently selected for frequency sweep measurements. Frequency sweeps (0.1–20 Hz, 1% strain, 25 °C) were performed within the LVR, and the storage modulus (G′), loss modulus (G″), and loss tangent (tan δ = G″/G′) were recorded.

### 2.5. Determination of Dough Stretching Properties

The stretching properties of the dough were determined using an extensograph (JMLD 150 Stable Micro Systems, Surrey, UK) with reference to the protocol described by Dahdah et al. [21]. The dough was prepared using a farinograph and shaped into cylindrical pieces (length: 15 cm; diameter: 3 cm). After resting in a controlled environment (30 °C, 85% relative humidity) for 45 min, each dough piece was clamped at both ends and stretched vertically until rupture. The stretching speed was 3.3 cm/s. The following parameters were recorded: tensile resistance, extensibility, stretch curve area, and stretch ratio.

### 2.6. Preparation of Biscuit

The preparation of biscuits was slightly modified based on the method reported by Kaderides et al. [22]. The biscuit dough formulation comprised: low-gluten wheat flour (100 g) supplemented with GSE at 0%, 0.2%, 0.4%, 0.6%, 0.8%, or 1% (*w*/*w*, relative to flour), butter (90 g), sucrose (40 g), and one fresh egg. Initially, the wheat flour was homogenized with a mixer (SM-201/202, SUPOR Home Appliances Co., Ltd., Hangzhou, China) for 30 s. Subsequently, butter and sucrose were added to the flour, and the mixture was continuously stirred at a low speed for approximately 5 min until uniformly mixed. After that, all the remaining ingredients were added to the mixer, which was then sealed to blend the mixture into a homogeneous dough for 15 min. After the mixing was completed, the obtained dough was transferred to a sealed container and allowed to rest at room temperature for 30 min to eliminate internal stress. For each formulation, three independent batches of dough were prepared. After resting, the dough was placed on a floured workbench and rolled into a uniformly thick 3-mm sheet using a rolling pin. Subsequently, the sheet was cut into 20-g rectangular pieces with dimensions of 50 mm (length) × 20 mm (width) using a stainless-steel biscuit cutter. Each dough piece weighed approximately 20 g. A total of 24 biscuits were produced per batch, with 6 biscuits placed on each baking tray (30 cm × 25 cm) lined with parchment paper. The electric oven (Midea PT3031, Wuhu Midea Kitchen and Bathroom Appliances Manufacturing Co., Ltd., Wuhu, China) was preheated to 210 °C for 15 min. The trays were placed on the middle rack, and the biscuits were baked at 210 °C for 10 min. Halfway through baking (at 5 min), the trays were rotated front-to-back to ensure uniform heat distribution. Post-baking, biscuits were allowed to cool at room temperature for 40 min, then stored in high-density polyethylene bags for subsequent characterization.

### 2.7. Determination of Color Properties of Biscuit

Chromatic attributes were evaluated according to the CIELAB system using diffuse reflectance spectrophotometry. A colorimeter (Linshang Technology, Shenzhen, China) was used to measure L*, a*, b*, and ΔE values under standardized illumination conditions. Measurements were performed using six independent samples [23].

### 2.8. Determination of the Baking Loss Rate of Biscuit

The baking loss rate of biscuits was calculated by weighing the samples before and after baking [24]. The calculation formula is as follows:

$$Baking\;loss\;rate\left(\%\right)=\frac{W0-W1}{W0}*100\%$$

Among them, W0 represents the initial weight of the biscuit dough. W1 represents the weight of the cooled biscuit.

### 2.9. Determination of the Diameter, Thickness, and Spread Factor of Biscuit

Each biscuit was measured at three equidistant points for diameter and at the center for thickness using vernier calipers. Mean values (mm) were calculated for each sample. Thickness was determined by stacking six biscuits and dividing the aggregate height by six. The diameter of each biscuit was measured with a vernier caliper (two measurements were taken by rotating the biscuit 90°). The spread factor of the biscuit was calculated as diameter/thickness [25]. All measurements were repeated 6 times. Measurements were performed using six independent samples.

### 2.10. Determination of the Textural Properties of Biscuit

Biscuit texture was analyzed following Masmoudi et al. [26]. Instrumental texture measurement was performed using a texture analyzer (TA-TX Plus, Stable Micro System, Godalming, Surrey, UK). The biscuits were cut into 1.0 cm squares to obtain uniform samples and minimize edge effects for texture profile analysis. The probe P/25 was selected, with a test speed of 2 mm/s, a strain of 60%, and a trigger force of 10 g. The samples were placed horizontally on the stage. A force-time graph was generated during the measurement, and textural parameters such as hardness, springiness, chewiness, and cohesiveness were acquired with the assistance of the software supplied with the instrument. Measurements were performed using six independent samples.

### 2.11. Preparation of Polyphenol Extract

The preparation of polyphenol extracts from biscuits was carried out with reference to the protocol described by Elkatry et al. [4], with minor adjustments made. Biscuit samples were ground into a fine powder using a laboratory mill and passed through a 60-mesh sieve. A 2.0-g portion of the powdered sample was mixed with 20 mL of methanol–water (70:30, *v*/*v*) in a 50-mL conical flask. The mixture was incubated on an orbital shaker at 150 rpm and 25 °C for 24 h. Subsequently, the mixture was centrifuged at 8000 rpm for 20 min (TGL-16G high-speed benchtop centrifuge, Shanghai Anting Scientific Instrument Co., Ltd., Shanghai, China). The supernatant was filtered through Whatman No. 41 filter paper and stored at 4 °C in amber vials until analysis.

### 2.12. Determination of TPC

TPC was quantified using a procedure from Abreu et al. [27], with minor methodological refinements. Specifically, 0.15 mL of the sample extract was mixed with 10-fold diluted Folin–Ciocalteu reagent (1.5 mL, 0.2 mol/L). After a 3-min pre-equilibration, 10 mL of a 7% sodium carbonate solution was added, and the mixture was incubated in the dark for 60 min. The absorbance at 785 nm was then recorded using a UV spectrophotometer (UPG-722, Beijing UPG General Technology Co., Ltd., Beijing, China). TPC values were expressed as mg gallic acid equivalents per gram dry weight (mg GAE/g DW).

### 2.13. Determination of TFC

TFC was determined following the method outlined by Sagar et al. [28]. 0.15 mL of the extract was mixed with 250 μL of a 1.6% sodium nitrite solution, and the mixture was kept in the dark for 5 min. Subsequently, 250 μL of a 3.2% (*m*/*v*) AlCl_3_ solution was added. After the mixture was allowed to stand for another 5 min, 250 μL of an 8% (*m*/*v*) sodium hydroxide solution was added to the mixture. The resulting mixture was stored in the dark at room temperature for 15 min, after which its absorbance was measured at 510 nm using the UV spectrophotometer. Results were presented as milligrams of rutin equivalents per gram of dry weight (RE mg/g DW). When the absorbance value fell outside the linear range, the extract was subjected to further dilution.

### 2.14. Determination of DPPH Free Radical Scavenging Rate

The determination of DPPH free radical scavenging rate was performed using the method described by De Camargo et al. [29] with slight modifications. Two milliliters of the sample extract were thoroughly mixed with 2 mL of 0.4 mmol/L DPPH^+^ solution. The mixture was placed in the dark at 25 °C for 30 min, and the absorbance of the sample reaction mixture (As) was measured at 517 nm. For the blank (control), 2 mL of absolute ethanol was substituted for the sample extract and mixed with 2 mL of DPPH^+^ solution; the absorbance of the blank (Ab) was measured under identical conditions. A sample blank (2 mL of sample extract mixed with 2 mL of absolute ethanol, without DPPH^+^) was also prepared to correct for background absorbance from the extract itself. The absorbance of the sample blank was negligible and thus was not incorporated into the final calculation. Three independent extractions were performed for each biscuit formulation, and each extract was analyzed in triplicate (n = 9). The DPPH radical scavenging rate was calculated as follows:

$$DPPH\;free\;radical\;scavenging\;rate\left(\%\right)=\frac{Ab-As}{Ab}*100\%$$

### 2.15. Determination of ABTS Free Radical Scavenging Rate

ABTS free radical scavenging rate was assessed using a procedure from De Camargo et al. [29], with slight methodological refinements. ABTS solution (7.4 mM) and potassium persulfate (2.6 mM) stock solutions were prepared. Equal aliquots of the two stock solutions were combined and allowed to react in the dark at room temperature for 12–16 h. Before use, the ABTS working solution was diluted with ethanol to an initial absorbance of 0.70 ± 0.02 at 723 nm. A 50 μL sample extract was combined with 450 μL ethanol, followed by the addition of 500 μL ABTS solution prepared immediately beforehand. After allowing 5 min for reaction stabilization, spectrophotometric absorbance was determined at 723 nm (A1). For the blank (control), 50 μL of absolute ethanol was substituted for the sample extract, and 450 μL of ethanol and 500 μL of diluted ABTS solution were added; the absorbance (A0) was measured using the same method. A sample blank (50 μL of sample extract mixed with 450 μL of ethanol and 500 μL of absolute ethanol, i.e., without ABTS was prepared to account for background absorbance. The absorbance of the sample blank was negligible (<0.05) and thus was not incorporated into the final calculation. Three independent extractions were performed for each formulation, and each extract was analyzed in triplicate (n = 9). The ABTS radical scavenging rate was calculated as follows:

$$ABTS\;free\;radical\;scavenging\;rate\left(\%\right)=\frac{A0-A1}{A0}*100\%$$

### 2.16. Sensory Evaluation

Twenty panelists (10 males and 10 females, aged 18–45 years, comprising faculty members and students from Qiqihar University) participated in the sensory appraisal of baked biscuits. Panelists were untrained consumers. Each biscuit was cut into two equal halves (approximately 2 g per portion) and served on a white ceramic plate at room temperature (approximately 25 °C). Samples were assigned random three-digit codes to eliminate bias. Panelists were instructed to rinse their mouths with room-temperature distilled water between samples, with a minimum inter-sample interval of 60 s. Panelists were directed to score each sample across five sensory dimensions, including flavor, texture, taste, color, and overall liking. Following Elketry et al. [4], a 9-point scoring scale was employed: 1 = extremely disliked, 2 = very disliked, 3 = moderately disliked, 4 = slightly disliked, 5 = neutral, 6 = slightly liked, 7 = moderately liked, 8 = very liked, 9 = extremely liked. The obtained scoring results were compiled and then subjected to statistical analysis.

### 2.17. Data Analysis

Data are presented as mean ± SD. Statistical analysis was performed using one-way analysis of variance (ANOVA) followed by Duncan’s multiple range test in Statistica v. 8.0 (Statsoft, Inc., Tulsa, OK, USA), with significance set at *p* < 0.05. The assumptions of normality and homogeneity of variance were verified before ANOVA. Pearson’s correlation analysis was performed to evaluate the associations among TPC, TFC, DPPH, and ABTS values, with statistical significance set at *p* < 0.05. For flour and dough properties, six independent measurements were performed for each formulation. For biscuit physical properties, three independent batches were prepared, with six biscuits randomly selected from each batch (n = 18). Antioxidant assays were performed using extracts from three independent batches, with triplicate measurements (n = 9).

## 3. Results and Discussion

### 3.1. Farinographic Properties of Low-Gluten Flour

Farinographic parameters serve as essential benchmarks for assessing how flour performs during processing. These parameters mirror dough behavior throughout mixing and downstream processing, forming the foundation for predicting both textural stability and processing tolerance in baked goods [30]. This method mainly includes six indicators. First, WA is determined by factors such as the molecular weight and structural conformation of amylose and amylopectin. This property is further shaped by the relative proportions of amylose and amylopectin, as well as the branching density and branch length distribution within these polysaccharides [31]. DT is governed by both gluten abundance and functional properties, alongside the flour’s hydration capacity. ST gauges the dough’s endurance under mechanical shear, with superior values signaling greater resistance to breakdown. The degree of softening, conversely, inversely mirrors gluten network integrity—lower readings correspond to a more resilient matrix. A higher DS value indicates that the dough is less capable of prolonged mechanical processing (over-mixing). FQN is a comprehensive evaluation index determined by a farinograph, which is used to quantify the overall quality and processing performance of flour [32]. The effects of different GSE addition levels on the farinographic properties of low-gluten flour are summarized in Table 1.

As shown in Table 1, no significant differences in WA were observed among all groups, indicating that GSE supplementation did not markedly affect the overall water absorption capacity of the low-gluten flour system. The DT initially decreased and subsequently increased with increasing GSE addition, with the shortest DT values observed in the 0.4% and 0.6% GSE groups. In contrast, the ST continuously increased with increasing GSE concentration. The 1.0% GSE group exhibited the longest ST, approximately eight times higher than that of the control, indicating enhanced resistance of the dough to mechanical mixing. The increased ST may be associated with interactions between GSE-derived phenolic compounds and gluten proteins. Non-covalent interactions, such as hydrogen bonding between phenolic hydroxyl groups and protein functional groups, may contribute to the stabilization of the gluten network [33]. The degree of softening (DS) initially decreased and subsequently increased with increasing GSE supplementation. The lowest DS value was observed in the 0.4% GSE group, suggesting reduced dough softening during mixing at moderate GSE levels. This effect may be related to the influence of phenolic compounds on gluten network behavior under appropriate supplementation conditions [34]. The FQN showed a similar concentration-dependent pattern, increasing initially and then decreasing, with the highest value observed at 0.4% GSE addition. This result indicates that moderate GSE supplementation improved the overall mixing quality and processing performance of low-gluten flour. However, excessive GSE addition may induce overly strong interactions between polyphenols and flour components, restricting gluten mobility and reducing the flexibility of the dough matrix, which may explain the decline in comprehensive dough quality at higher supplementation levels. Similar effects were reported by Tolve et al. [35], who found that grape pomace powder supplementation increased dough stability time.

Collectively, moderate GSE supplementation (0.4–0.6%) improved dough mixing stability and reduced softening during mixing, whereas excessive addition resulted in excessive reinforcement of the dough structure and provided no further improvement in overall processing performance, indicating a concentration-dependent effect of GSE. These results suggest that GSE influences dough development and mechanical stability; therefore, its effects on starch gelatinization behavior were further evaluated by RVA analysis.

### 3.2. Gelatinization Properties of Low-Gluten Flour

The gelatinization of starch is characterized by the penetration of water into the crystalline regions of starch granules, resulting in the transition of starch from an ordered to a disordered structure during heating. This process provides important information on the thermal behavior and viscoelastic characteristics of flour systems [36]. The gelatinization properties mainly include peak viscosity, gelatinization temperature, breakdown value, and setback value. Peak viscosity represents the maximum viscosity reached during heating and reflects the swelling capacity of starch granules before structural disruption [37]. Gelatinization temperature indicates the temperature at which starch granules begin to lose their crystalline structure. The breakdown value reflects the stability of swollen starch granules during continuous heating and mechanical shear, whereas the setback value is commonly used to evaluate viscosity recovery during cooling and the short-term reassociation tendency of starch molecules [38,39].

**Figure 1:**
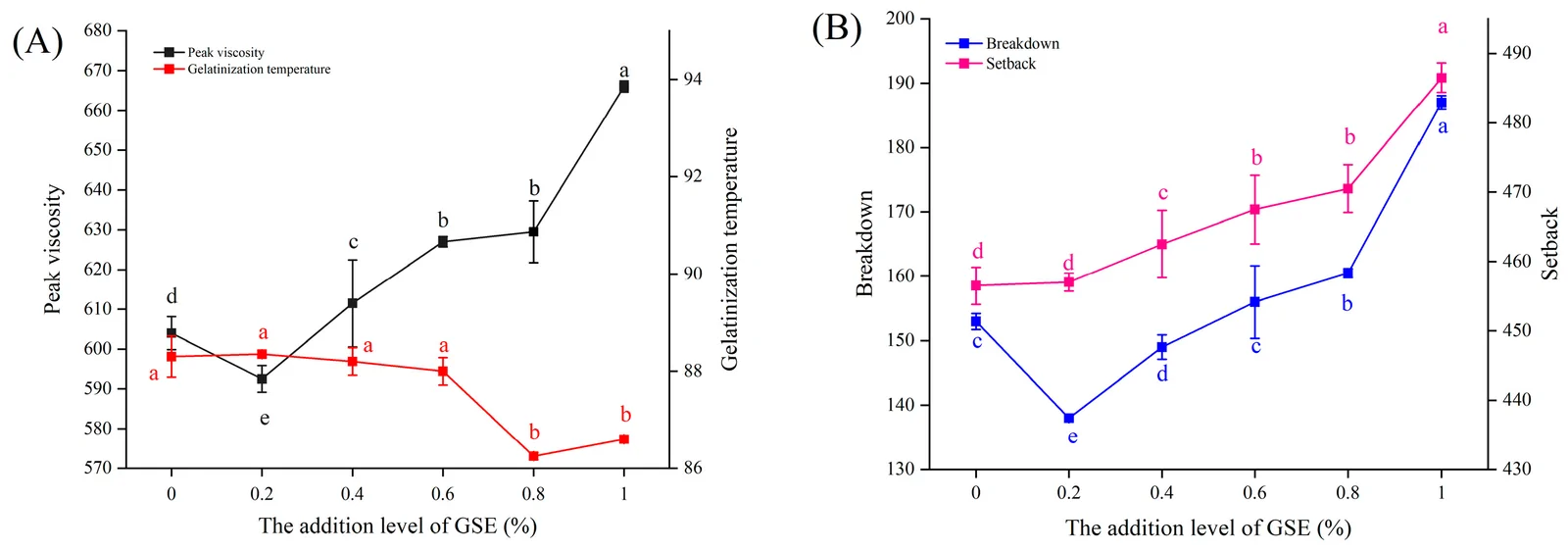
Effects of GSE addition levels on gelatinization properties of low-gluten flour. Note: Different lowercase letters indicate significant differences; (**A**): Peak viscosity and Gelatinization temperature; (**B**): Breakdown and Setback.

The effects of different GSE addition levels on the gelatinization properties of low-gluten flour are shown in Figure 1. As the GSE concentration increased, peak viscosity initially decreased and subsequently increased (Figure 1A), with the highest value observed at 1.0% GSE supplementation. These results indicate that GSE affected starch swelling behavior in a concentration-dependent manner. The initial reduction in peak viscosity may be attributed to interactions between GSE polyphenols and water, where the hydroxyl groups of polyphenols compete with starch molecules for available water, thereby limiting starch granule swelling during heating [40]. As shown in Figure 1B, the breakdown value decreased markedly at 0.2% GSE addition and then gradually increased with further supplementation. The lowest breakdown value, representing a 10.9% reduction compared with the control, indicates enhanced stability of swollen starch granules under heating and shear conditions [41]. This improvement may be associated with interactions between GSE polyphenols and starch molecules. Phenolic hydroxyl groups can interact with starch chains through hydrogen bonding, which may restrict starch molecular mobility, regulate chain rearrangement, and enhance the resistance of swollen starch granules to thermal and mechanical disruption [42]. However, excessive GSE addition may result in stronger interactions between polyphenols and starch components, which could excessively restrict starch chain mobility and affect the flexibility of the starch–flour matrix during processing. In contrast, the setback value increased continuously with increasing GSE concentration, suggesting enhanced viscosity recovery during the cooling stage [2]. This phenomenon may be related to the regulation of starch molecular reassociation by GSE polyphenols during cooling. Polyphenol–starch interactions may influence starch chain rearrangement and water distribution, thereby affecting short-term viscosity recovery [42]. However, it should be noted that RVA setback primarily reflects viscosity recovery during the cooling cycle and does not directly represent long-term starch retrogradation. Together with the farinographic results, these findings suggest that moderate GSE supplementation may simultaneously improve dough mixing stability and starch paste stability, thereby contributing to favorable processing properties of low-gluten flour.

### 3.3. The Dynamic Rheological Properties of Low-Gluten Flour

**Figure 2:**
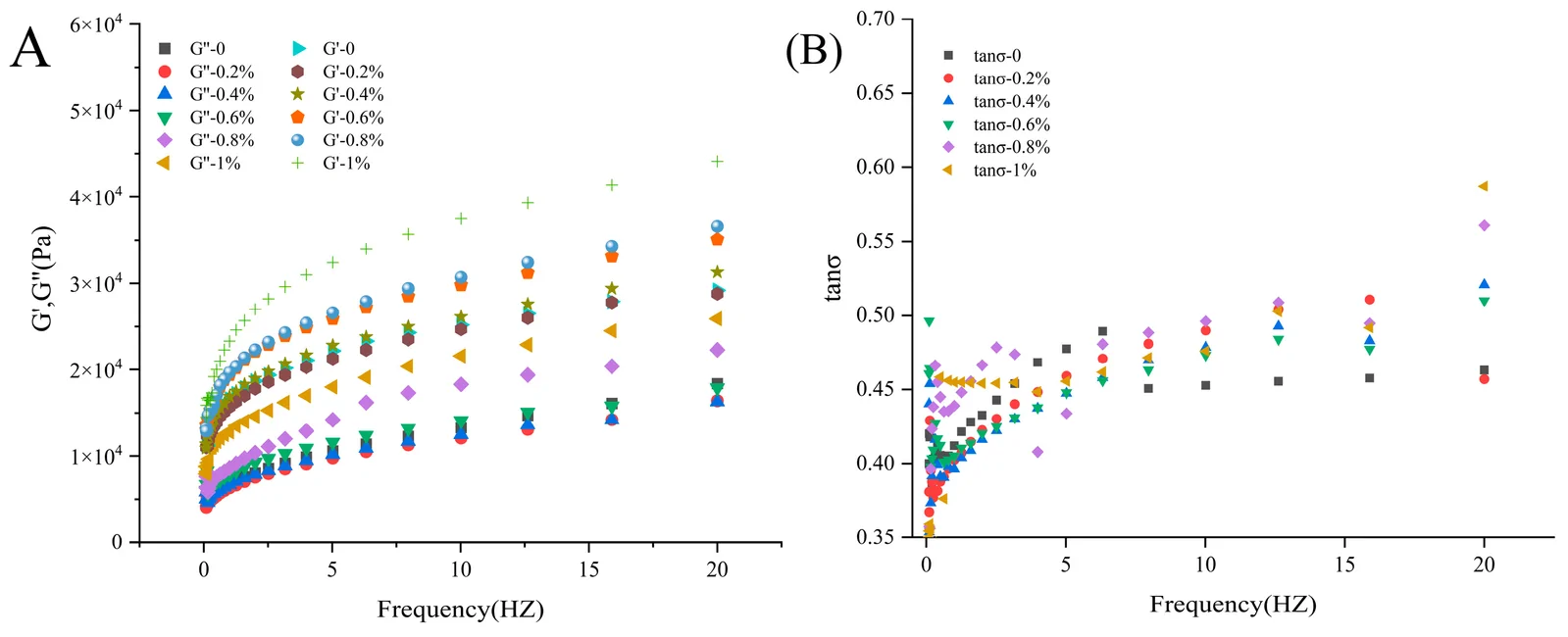
Effects of GSE addition levels on dynamic rheological properties of low-gluten flour. (**A**): G′ and G″; (**B**): tan δ.

Dynamic rheological properties provide important information regarding the viscoelastic behavior and structural characteristics of dough, which are closely related to dough processing performance and the quality of baked products [43]. The dynamic rheological spectrum is generally described using three fundamental parameters: the G′, which represents the elastic (solid-like) response of dough; the G″, which reflects its viscous (liquid-like) behavior; and the tan δ, which describes the balance between elasticity and viscosity under oscillatory deformation [44]. The effects of different GSE addition levels on the dynamic rheological properties of low-gluten flour dough are shown in Figure 2.

Figure 2A shows that both G′ and G″ increased with increasing oscillation frequency, demonstrating the typical frequency-dependent viscoelastic behavior of wheat dough systems. This behavior reflects the dynamic response of dough macromolecules, primarily gluten proteins and starch molecules, which undergo molecular rearrangement and relaxation under different deformation timescales [45]. Across the entire frequency range, G′ remained higher than G″, indicating that the elastic component dominated the viscoelastic behavior of all dough samples.

At lower frequencies, the G′ and G″ values of different groups were relatively similar, whereas at frequencies above 10 Hz, GSE-supplemented doughs, particularly the 1.0% GSE group, exhibited higher G′ and G″ values than the control. As shown in Figure 2B, the tan δ values of both the control and GSE-treated groups gradually decreased with increasing oscillation frequency, and all values remained below 1.0, indicating that the elastic component dominated the viscoelastic behavior of all dough samples. Moreover, the lower tan δ values observed in GSE-supplemented doughs suggest that GSE incorporation may further enhance the elastic contribution of the dough structure, particularly at higher deformation frequencies [46]. Similar rheological changes have been reported for wheat dough supplemented with other polyphenol-rich ingredients, such as apple pomace [2]. However, differences in composition, especially dietary fiber content, mean that such effects cannot be directly compared with purified GSE.

Although the 1.0% GSE group showed the highest G′ and G″ values, increased viscoelasticity does not necessarily represent improved dough processability. Excessive reinforcement of the dough structure may reduce flexibility and deformation capacity. This phenomenon may be associated with stronger interactions between GSE polyphenols and flour components at higher supplementation levels, which could restrict molecular mobility and limit the rearrangement of the dough matrix during deformation. In contrast, moderate GSE supplementation (0.2–0.6%) resulted in a more balanced viscoelastic profile, which was consistent with the favorable farinograph and RVA characteristics. The specific molecular interactions responsible for these rheological changes require further structural investigation.

### 3.4. Stretching Properties of Low-Gluten Flour

**Figure 3:**
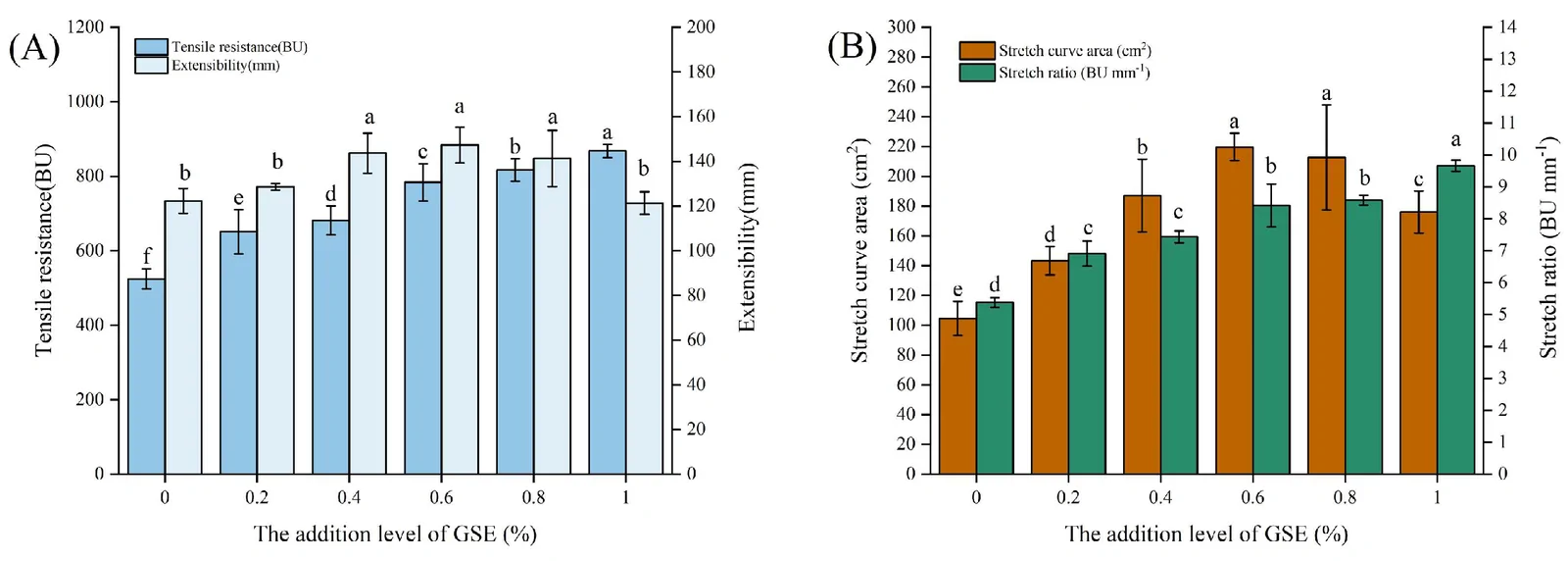
Effects of GSE addition levels on dough extensibility properties of low-gluten flour. Note: Different lowercase letters indicate significant differences. Note: Different lowercase letters indicate significant differences; (**A**): tensile resistance and extensibility; (**B**): stretch curve area and stretch ratio.

Stretching properties, including tensile resistance, extensibility, stretch curve area, and stretch ratio, are important indicators for evaluating the mechanical behavior of dough during processing, such as shaping and baking. Tensile resistance represents the maximum force required to deform or break the dough, whereas extensibility reflects the ability of the dough to undergo deformation without rupture [47]. The stretch curve area represents the energy required during stretching, while the stretch ratio provides information regarding the balance between dough resistance and extensibility [48]. Figure 3 illustrates the effects of different GSE addition levels on the stretching properties of low-gluten flour.

As shown in Figure 3A, tensile resistance gradually increased with increasing GSE concentration. At 1.0% GSE addition, tensile resistance reached the highest value, representing a 51.8% increase compared with the control. In contrast, dough extensibility initially increased and subsequently decreased with increasing GSE supplementation. The maximum extensibility was observed at 0.6% GSE, showing a 10.3% increase compared with the control. Similarly, the stretch curve area increased initially and then decreased, reaching the highest value at 0.6% GSE addition (Figure 3B). The stretch ratio continuously increased with increasing GSE concentration, with the highest value observed at 1.0% GSE. These results indicate that moderate GSE supplementation (0.2–0.6%) improved the balance between dough strength and extensibility, whereas excessive GSE addition increased dough resistance while reducing deformation capacity. The increased tensile resistance at higher GSE levels may be related to interactions between grape seed polyphenols and wheat gluten proteins, which may influence gluten network behavior. Previous studies have suggested that polyphenols can interact with gluten proteins through non-covalent interactions, including hydrogen bonding and hydrophobic interactions, thereby affecting gluten functionality [49]. However, the specific molecular interactions involved require further investigation. At excessive supplementation levels, stronger interactions between polyphenols and flour components, together with increased competition for water, may restrict gluten mobility and reduce dough extensibility. The optimal stretching performance observed at 0.6% GSE was consistent with the favorable rheological behavior, suggesting that moderate GSE addition improved dough structural stability while maintaining sufficient flexibility. Similar effects have been reported for other botanical extracts; for example, Sokolova [50] found that optimized hop extract supplementation improved dough strength and tensile resistance.

When GSE addition reached 0.8% or higher, the combined changes observed in farinographic properties (increased DS and reduced FQN), rheological behavior (increased G′ and G″ with decreased tan δ), and stretching parameters (reduced extensibility and stretch curve area) indicated the formation of a stronger but less flexible dough structure. Although excessive GSE enhanced dough resistance, the excessive reinforcement of the dough matrix likely restricted deformation ability during processing and baking, which may contribute to the increased hardness observed in biscuits.

### 3.5. Color of Biscuit

Among the sensory attributes of biscuits, color plays an important role in consumer acceptance and quality perception [51]. Color evaluation is commonly performed using the CIELAB system, including the parameters L*, a*, b*, and total color difference (ΔE). The L* value represents lightness (0 = black, 100 = white), whereas a* and b* indicate chromatic coordinates ranging from green (−) to red (+) and blue (−) to yellow (+), respectively [40]. ΔE represents the overall color difference between each sample and the control biscuit without GSE supplementation. According to commonly used CIELAB criteria, ΔE values below 2 are generally considered difficult to distinguish visually, whereas values above 5 usually represent clearly perceptible color differences [52].

Table 2 summarizes the color characteristics of biscuits supplemented with different levels of GSE. The L* value gradually decreased with increasing GSE concentration, indicating a darker appearance of the biscuits. The control biscuit exhibited the highest L* value, whereas the 1.0% GSE group showed the lowest value. This reduction in lightness was mainly attributed to the inherent reddish-brown color of GSE powder. Moreover, thermal processing and interactions between GSE-derived compounds and food matrix components may have further influenced color development during baking [3]. The a* value increased progressively with increasing GSE addition, from 3.86 in the control to 7.75 in the 1.0% GSE group, whereas the b* value decreased from 28.94 to 17.41. All biscuits exhibited positive a* and b* values, indicating predominant reddish and yellowish tones. The gradual shift toward a darker reddish-yellow appearance was mainly associated with the intrinsic color of GSE, while phenolic compounds and their oxidation products may also have contributed to color formation during baking [4]. The ΔE values increased with increasing GSE concentration, indicating that GSE incorporation induced visually distinguishable color changes compared with the control biscuit. Moderate GSE supplementation produced an acceptable color modification that was consistent with the higher sensory acceptance observed at 0.6% GSE addition, whereas excessive supplementation resulted in a darker appearance that may negatively affect consumer perception.

### 3.6. Baking Loss Rate of Biscuit

**Figure 4:**
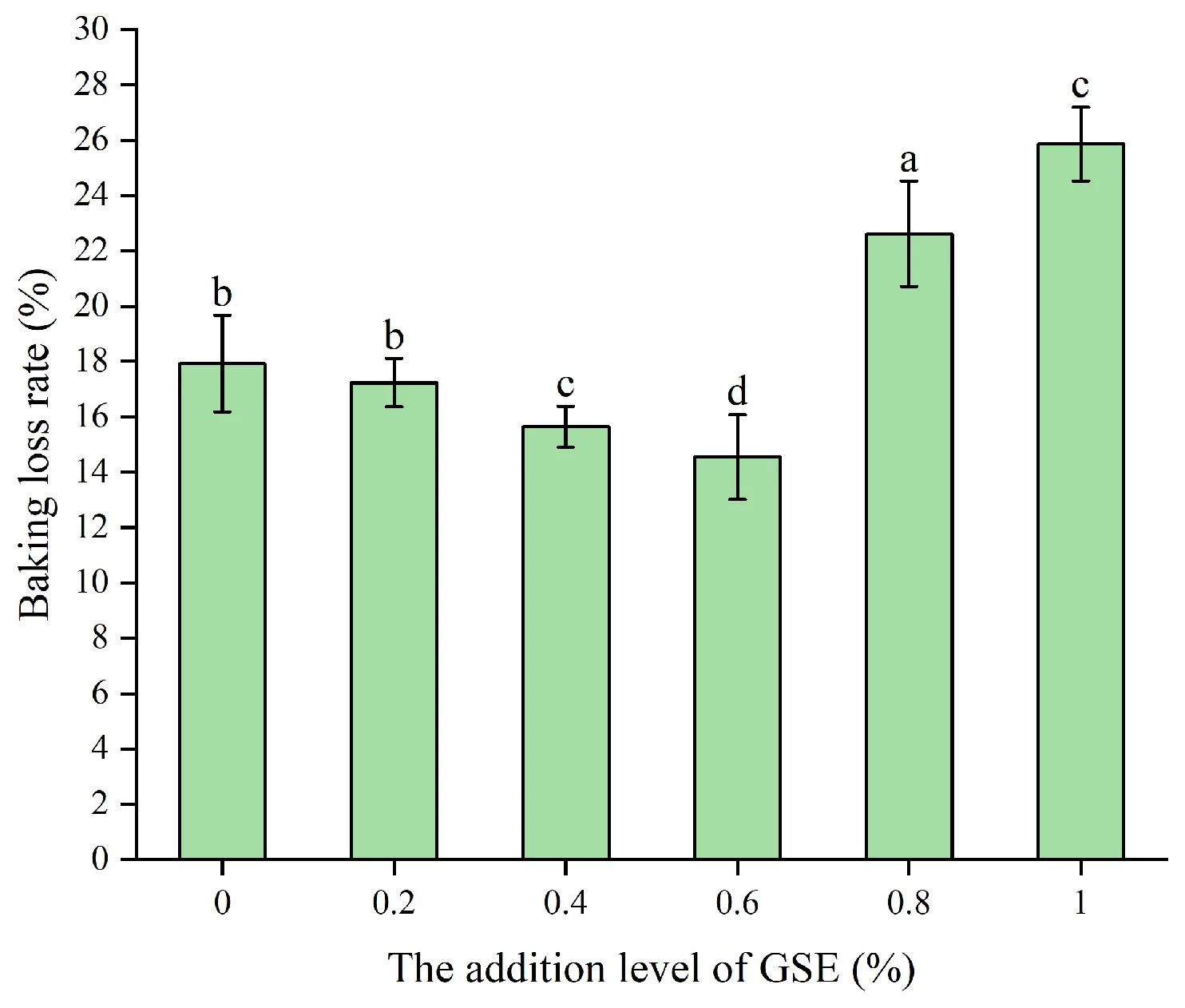
Effects of GSE addition levels on the baking loss rate of biscuits. Note: Different lowercase letters indicate significant differences.

Baking loss represents the decrease in sample weight during baking, which is mainly attributed to moisture evaporation under high-temperature conditions. Additionally, the melting and partial volatilization of butter, as well as the release of low-molecular-weight volatile compounds generated during thermal treatment, may also contribute to overall weight reduction [53]. As baking loss is closely associated with water retention capacity during baking, it can significantly affect biscuit texture, appearance, and overall quality. Figure 4 presents the effects of different GSE addition levels on the baking loss of biscuits.

A non-linear dose-dependent effect of GSE supplementation on baking loss was observed, with the lowest value obtained at 0.6% GSE addition, representing a 16.7% reduction compared with the control group. At an appropriate concentration, GSE polyphenols may enhance the structural integrity of the dough matrix through non-covalent interactions, including hydrogen bonding and hydrophobic interactions, with gluten proteins. Meanwhile, interactions between polyphenols and starch molecules may further contribute to the reinforcement of the gluten–starch network. These interactions could improve the stability of the three-dimensional dough structure and enhance its water-holding capacity, thereby reducing moisture loss during baking. However, when the GSE concentration exceeded 0.6%, excessive polyphenols may disturb the balance of gluten–starch interactions and compete for water-binding sites, thereby limiting the formation of an optimal dough network and increasing moisture loss during baking [54,55]. Notably, the lowest baking loss observed in the 0.6% GSE group coincided with its maximum extensibility and favorable rheological behavior, suggesting that moderate GSE supplementation optimized dough network formation by balancing structural strength and flexibility, thereby improving both water retention and baking performance. Similar findings were reported by Koh et al. [51], who demonstrated that biscuits supplemented with an appropriate amount of waste tea leaf powder exhibited significantly reduced baking loss compared with control biscuits.

### 3.7. Diameter, Thickness, and Spread Factor of Biscuit

Biscuit diameter, thickness, and spread factor serve as critical metrics for gauging expansion extent, texture uniformity, and visual appeal to consumers throughout the baking process. The spread factor (diameter/thickness) is one of the important physical parameters for assessing biscuit quality, as it is related to the texture and overall mouthfeel of biscuits. An appropriate spread factor is generally associated with desirable biscuit texture and consumer acceptance [51,56].

As shown in Table 3, GSE supplementation significantly influenced biscuit diameter, thickness, and spread factor (*p* < 0.05). With increasing GSE addition, biscuit diameter gradually increased and reached the highest value at 1.0% GSE supplementation, showing a 5.9% increase compared with the control group. Previous studies have indicated that biscuit diameter is closely related to dough strength and its ability to resist deformation during baking [57]. In contrast, biscuit thickness exhibited a continuous decreasing trend with increasing GSE concentration. The 0.8% and 1.0% GSE groups showed the lowest thickness values (12.0 mm), corresponding to a 33.3% reduction compared with the control. Accordingly, the spread factor increased progressively with GSE supplementation and reached its maximum value at 1.0% GSE, which was 20.8% higher than that of the control. The enhanced spread factor may be attributed to GSE-induced modifications in dough rheological properties and water distribution, which reduced the resistance of dough to lateral expansion during baking [58]. Furthermore, interactions between GSE polyphenols and flour macromolecules, including gluten proteins and starch, may alter the internal dough structure and promote matrix rearrangement, thereby facilitating biscuit expansion during baking. Similar results were reported by Koh et al. [51], who found that partial substitution of wheat flour with waste tea leaf powder significantly increased the spread factor of biscuits. Likewise, Sik et al. [59] demonstrated that rose petal supplementation progressively enhanced the spread factor of biscuits.

It is noteworthy that the spread factor results were consistent with the baking loss findings. Although the highest spread factor was observed at 1.0% GSE addition, the lowest baking loss occurred at 0.6% GSE supplementation, indicating that different GSE levels influenced distinct aspects of biscuit formation. Moderate GSE addition favored water retention and maintained a balanced dough structure, whereas excessive supplementation promoted greater spreading behavior but was accompanied by reduced dough flexibility and increased hardness. Therefore, the overall quality improvement of biscuits depended on the balance between expansion, moisture retention, texture, and sensory acceptance rather than a single physical parameter.

### 3.8. Textural Properties of Biscuit

**Figure 5:**
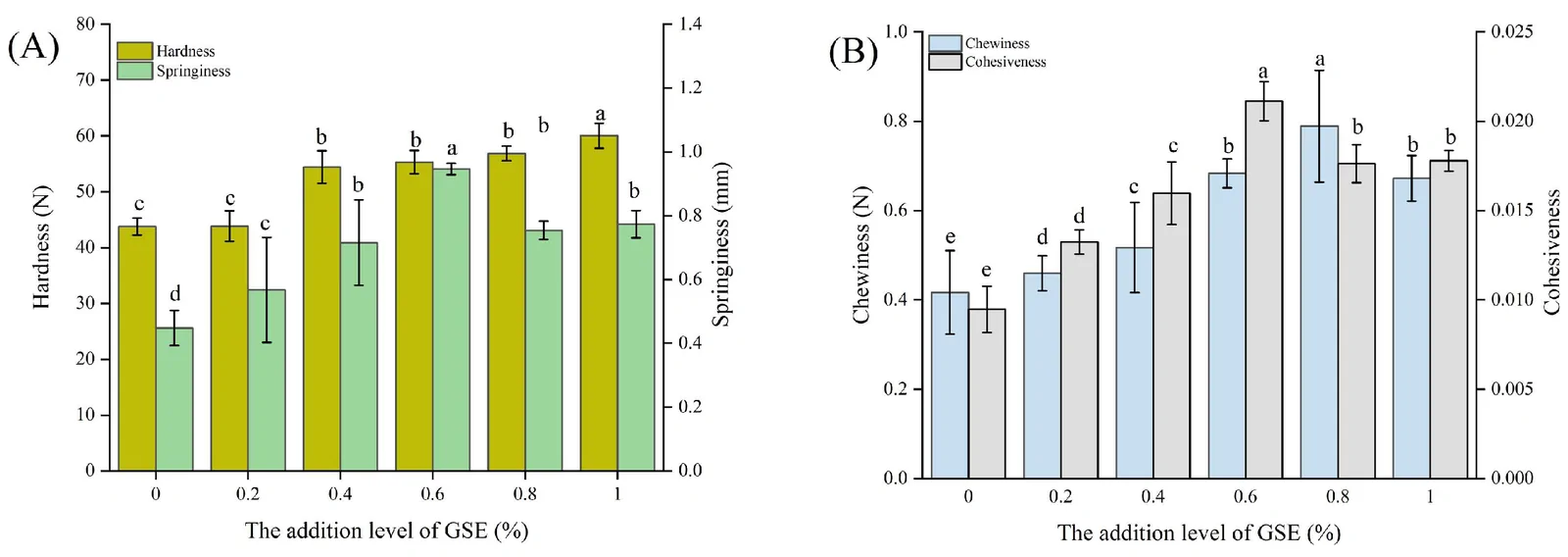
Effects of GSE addition levels on textural properties of biscuits. Note: Different lowercase letters indicate significant differences. Note: Different lowercase letters indicate significant differences; (**A**): hardness and springiness; (**B**): chewiness and cohesiveness.

Textural properties, including hardness, springiness, chewiness, and cohesiveness, are critical indicators of biscuit quality and consumer acceptability. Hardness represents the force required to deform a biscuit, whereas springiness reflects its ability to recover after compression. Chewiness describes the energy required during mastication, and cohesiveness indicates the integrity of the internal biscuit structure [60]. The effects of different GSE addition levels on biscuit textural properties are shown in Figure 5.

The 1.0% GSE group exhibited the highest hardness, which was 34.9% higher than that of the control (Figure 5A). Previous studies have shown that biscuit hardness is closely related to the development of the biscuit matrix during baking, particularly its porosity and matrix strength [61]. Therefore, the increased hardness observed at 1.0% GSE may be associated with GSE-induced changes in dough rheological behavior and moisture distribution, which could influence the formation of the biscuit matrix during baking [62,63]. However, because the internal microstructure was not directly examined in the present study, this interpretation requires further confirmation. The 0.6% GSE group showed the highest springiness, representing a 110.2% increase compared with the control. However, springiness decreased when GSE addition increased to 0.8–1.0%, which may be related to excessive polyphenol incorporation altering the dough matrix and reducing the elastic recovery of the baked biscuit. As shown in Figure 5B, chewiness and cohesiveness exhibited similar concentration-dependent trends, with both parameters increasing initially and subsequently decreasing. The highest chewiness was observed at 0.8% GSE, reaching 1.90-fold that of the control, whereas cohesiveness reached its maximum at 0.6% GSE, corresponding to a 2.21-fold increase. The increased chewiness was consistent with the higher hardness observed at elevated GSE levels, while the enhanced cohesiveness at moderate GSE concentrations suggested improved structural integrity of the biscuit matrix.

Overall, moderate GSE supplementation improved biscuit textural characteristics by enhancing springiness, cohesiveness, and chewiness while avoiding excessive hardness. These results were consistent with the improved dough extensibility and reduced baking loss observed at appropriate GSE levels, indicating that moderate GSE addition promoted a favorable balance between structural integrity and textural quality. Among the tested concentrations, 0.6% GSE provided the most favorable balance between biscuit hardness, elasticity, cohesiveness, and overall quality. In contrast, excessive GSE supplementation increased hardness and reduced elasticity, which may be associated with excessive reinforcement of the dough matrix caused by stronger interactions between GSE polyphenols and flour components. The underlying molecular mechanisms require further investigation. Similar increases in biscuit hardness have also been reported following the incorporation of pumpkin powder [62] and spent tea powder [51].

### 3.9. TPC, TFC, and DPPH and ABTS Radical Scavenging of Biscuits

**Figure 6:**
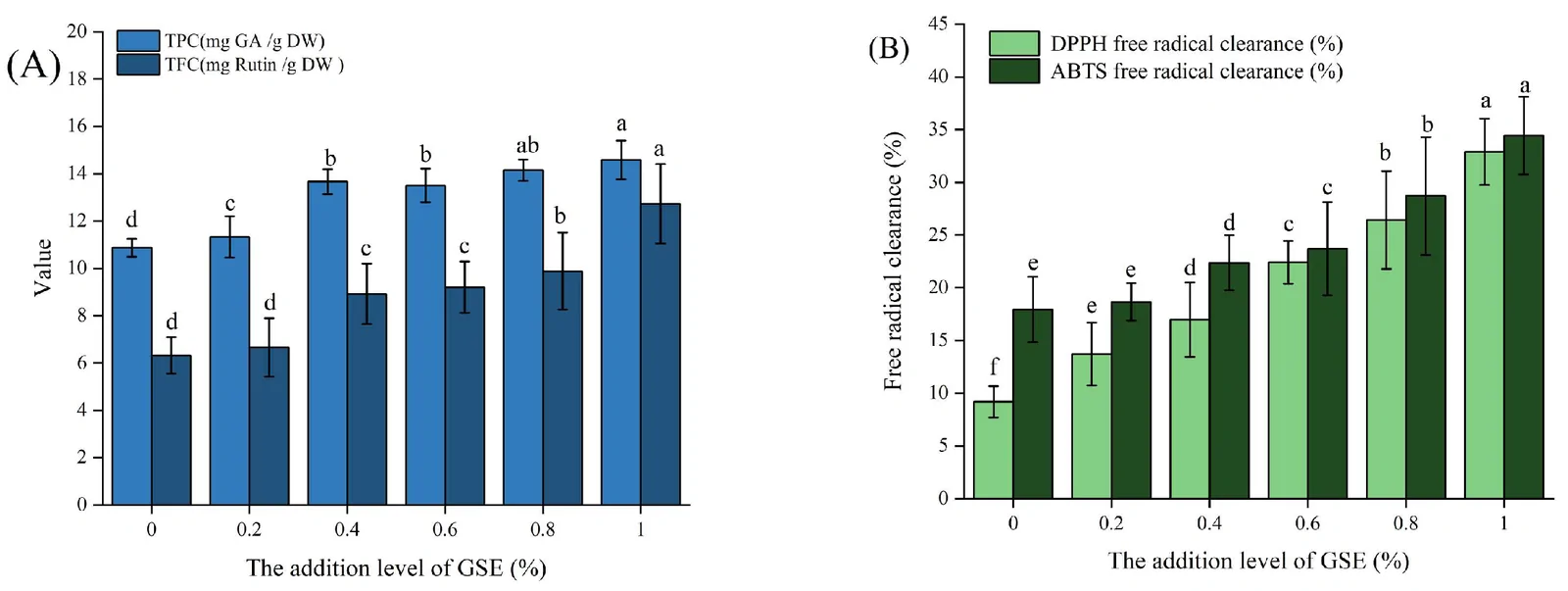
Effects of GSE addition levels on TPC, TFC, and DPPH and ABTS radical scavenging of biscuits. Note: Different lowercase letters indicate significant differences. (**A**): TPC and TFC; (**B**): DPPH and ABTS radical clearance.

The antioxidant properties of the product could be assessed by measuring total phenolic content (TPC), total flavonoid content (TFC), and DPPH and ABTS radical scavenging assays. The results are shown in Figure 6.

As shown in Figure 6A, both TPC and TFC increased progressively with increasing GSE supplementation. At 1.0% GSE addition, the TPC and TFC of biscuits reached their maximum values, representing increases of 32% and 75%, respectively, compared with the control. These increases were mainly attributed to the incorporation of phenolic-rich GSE. However, the measured values represent extractable phenolic and flavonoid compounds after baking rather than the total amount initially added. This is because during baking, phenolic compounds may undergo thermal degradation, oxidation, or interactions with food matrix components, which could reduce their extractability or alter their antioxidant activity. As shown in Figure 6B, control biscuits also exhibited measurable antioxidant activity, which may be associated with naturally occurring antioxidant compounds in the ingredients and antioxidant-active compounds generated during baking. Previous studies have suggested that Maillard reaction products, such as melanoidins, may possess radical-scavenging activity [4]. Both DPPH and ABTS radical-scavenging activities increased significantly with increasing GSE concentration, indicating enhanced in vitro antioxidant capacity of GSE-fortified biscuits. However, the highest antioxidant capacity observed at 1.0% GSE does not necessarily indicate the best overall product quality. The practical application of GSE in biscuits requires balancing functional enhancement with processing performance and consumer acceptance.

**Figure 7:**
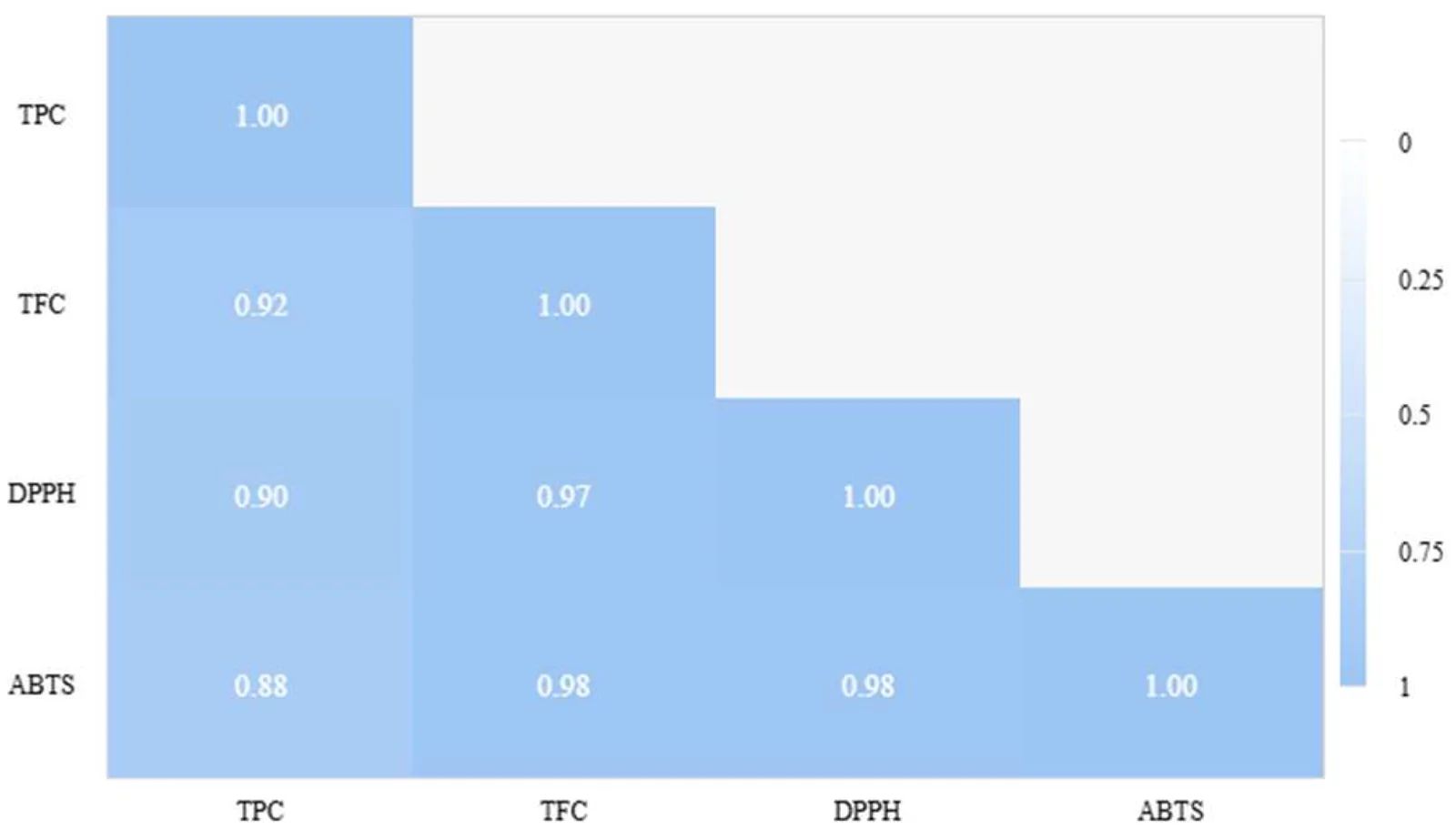
Pearson correlation heatmap of TPC, TFC, DPPH radical scavenging activity, and ABTS radical scavenging activity of GSE-fortified biscuits. The color gradient indicates the strength of the correlation coefficient (r), with darker red representing stronger positive correlations.

Pearson correlation analysis was further performed to investigate the relationships among TPC, TFC, DPPH, and ABTS values (Figure 7). Strong positive correlations were observed among all measured parameters, with correlation coefficients ranging from 0.88 to 0.98 (*p* < 0.01). TPC showed positive correlations with TFC (r = 0.92), DPPH (r = 0.90), and ABTS (r = 0.88), while TFC exhibited stronger correlations with DPPH (r = 0.97) and ABTS (r = 0.98). Moreover, DPPH and ABTS activities were highly correlated (r = 0.98), consistent with their similar concentration-dependent trends. These results suggest that GSE-derived phenolic and flavonoid compounds were major contributors to the increased radical-scavenging activity of fortified biscuits. Nevertheless, these correlations indicate association rather than direct causation, and other antioxidant-active components may also contribute.

DPPH and ABTS assays provide useful comparative information on chemical antioxidant capacity but cannot fully represent antioxidant behavior in complex biological systems. Further studies involving simulated digestion or biological evaluation are needed to better understand the functional effects of GSE-fortified biscuits. Previous studies using UPLC–MS/MS have identified phenolic acids, proanthocyanidins, flavonoids, flavan-3-ols, and stilbenes as major constituents of GSE [64]. Thus, the enhanced antioxidant activity of GSE-fortified biscuits was likely mainly associated with the introduced phenolic compounds, while endogenous compounds in the ingredients and baking-derived antioxidant products may also have contributed. Similar increases in TPC, TFC, and antioxidant activity have been reported in bread supplemented with grape-derived ingredients [4] and biscuits incorporated with coffee silverskin powder [7].

### 3.10. Sensory Evaluation

**Figure 8:**
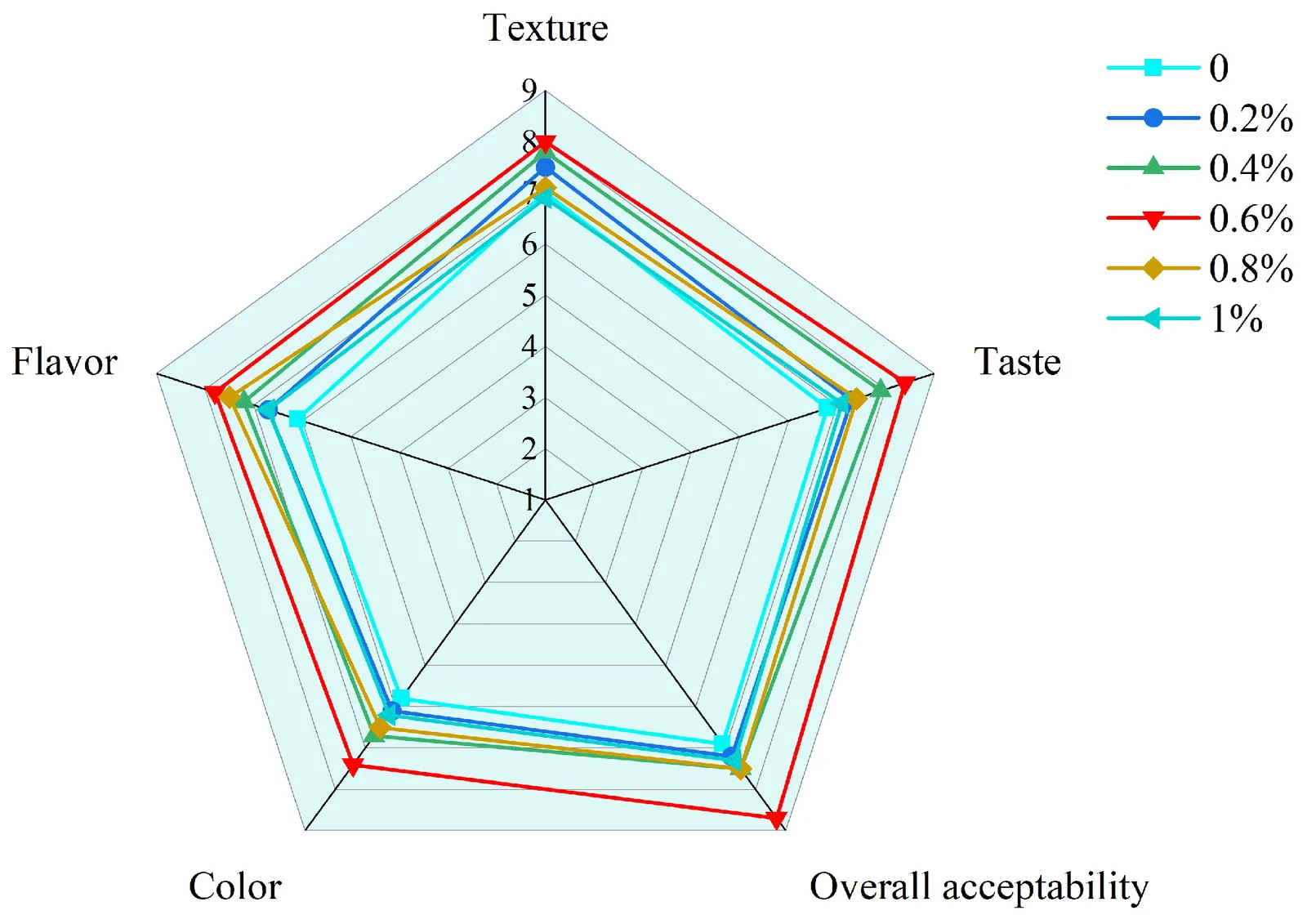
Effects of GSE addition levels on sensory evaluation of biscuits.

Sensory evaluation, including texture, flavor, taste, color, and overall acceptability, is an important parameter for assessing consumer acceptance and the potential application of biscuits [23]. Figure 8 presents the effects of different GSE addition levels on the sensory attributes of biscuits.

As shown in Figure 8, control biscuits exhibited the lowest overall sensory scores among all treatments. Texture, flavor, and taste scores initially increased and subsequently decreased with increasing GSE concentration, with the highest values observed at 0.6% GSE supplementation. The improved flavor and taste acceptance at moderate GSE levels may be associated with the incorporation of GSE-derived compounds into the biscuit matrix, whereas excessive addition may negatively affect sensory perception. Similarly, biscuits supplemented with 0.6% GSE showed significantly higher color scores (7.4) than the control (5.5). The highest overall acceptability score was observed at 0.6% GSE, confirming that moderate supplementation provided the most favorable sensory profile. In contrast, excessive GSE addition reduced sensory acceptance, likely due to the increased hardness and greater color differences observed at higher GSE levels. The agreement between instrumental measurements and sensory evaluation further supports the beneficial effects of moderate GSE supplementation. The moderate color difference observed for 0.6% GSE biscuits (Table 2) corresponded well with their higher sensory color scores, while the improved springiness and cohesiveness determined instrumentally (Figure 5) were consistent with the higher sensory texture scores. Conversely, 1.0% GSE biscuits exhibited the highest hardness and greatest color difference, accompanied by reduced sensory scores. These results suggest that moderate GSE addition achieved a balance between technological properties and sensory quality.

From an application perspective, 0.6% GSE supplementation provided the most favorable balance among dough performance, biscuit quality, antioxidant enhancement, and sensory acceptance. Although higher GSE concentrations further increased phenolic contents and in vitro antioxidant activity, excessive supplementation negatively affected certain technological and sensory attributes, including dough flexibility, biscuit hardness, and color perception. The observed technological improvements suggest that moderate GSE addition may facilitate dough handling by enhancing stability and maintaining balanced viscoelasticity, while improved extensibility and reduced baking loss may contribute to better structural integrity and texture retention of biscuits. Therefore, controlling GSE supplementation level is critical for achieving a balance between functional enhancement and technological performance in bakery applications.

## 4. Conclusions

This study demonstrated the concentration-dependent effects of grape seed extract (GSE) supplementation on dough functionality and biscuit quality. Moderate GSE addition improved dough stability, reduced dough softening, and achieved a favorable balance between dough strength and extensibility, while enhancing biscuit texture, sensory acceptance, phenolic contents, and in vitro antioxidant activity. However, excessive GSE supplementation increased biscuit hardness and reduced dough extensibility, indicating that higher GSE levels did not continuously improve overall product quality. Among the tested concentrations, 0.6% GSE provided the most favorable technological balance between dough processability, biscuit texture, sensory quality, and antioxidant enhancement.

These findings reveal that the functionality of GSE-fortified bakery products depends on balancing bioactive enrichment with dough processing performance rather than simply increasing GSE concentration. The observed effects may be associated with interactions between GSE polyphenols and flour components, although the underlying molecular mechanisms require further investigation. From an application perspective, this study provides practical guidance for developing GSE-fortified biscuits by identifying an appropriate supplementation level that improves nutritional functionality while maintaining processing performance and consumer acceptance. Future studies should employ structural characterization techniques, simulated digestion or biological evaluation, and storage stability analysis to further clarify polyphenol–flour interactions and evaluate the long-term quality and functional effects of GSE-enriched bakery products.

## Figures and Tables

**Table 1:** Effects of GSE addition levels on farinograph properties of low-gluten flour.

GSE Addition Levels	WA (%)	DT (min)	ST (min)	DS (FU)	FQN
0	52.0 ± 0.1 ^a^	1.7 ± 0.10 ^a^	2.4 ± 0.21 ^e^	55.3 ± 2.5 ^b^	29.7 ± 1.5 ^c^
0.2%	51.8 ± 0.12 ^a^	1.6 ± 0.23 ^a^	9.1 ± 0.46 ^d^	45.7 ± 3.5 ^c^	32.0 ± 1.0 ^b^
0.4%	52.2 ± 0.42 ^a^	1.5 ± 0.11 ^a^	12.7 ± 2.1 ^c^	37.7 ± 9.8 ^e^	40.7 ± 4.7 ^a^
0.6%	51.9 ± 0.23 ^a^	1.5 ± 0.20 ^a^	16.1 ± 0.86 ^b^	43.0 ± 3.6 ^d^	31.3 ± 7.5 ^b^
0.8%	52.2 ± 0.36 ^a^	1.6 ± 0.16 ^a^	16.7 ± 1.2 ^b^	49.3 ± 9.6 ^a^	24.3 ± 1.5 ^d^
1%	52.1 ± 0.26 ^a^	1.6 ± 0.35 ^a^	18.9 ± 1.1 ^a^	50.0 ± 7.8 ^b^	20.0 ± 6.2 ^e^

Note: Different lowercase letters indicate significant differences.

**Table 2:** Effects of GSE addition levels on the color properties of biscuit.

GSE Addition Levels	L*	a*	b*	∆E	Pictures
0	70.43 ± 0.31 ^a^	3.86 ± 1.41 ^d^	28.94 ± 1.30 ^a^	/	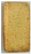
0.2%	67.17 ± 0.75 ^b^	5.12 ± 0.37 ^c^	23.53 ± 1.40 ^b^	8.11 ± 0.29 ^e^	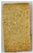
0.4%	65.18 ± 1.10 ^c^	5.47 ± 0.36 ^c^	20.73 ± 1.01 ^c^	9.29 ± 1.17 ^d^	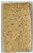
0.6%	65.47 ± 0.86 ^c^	6.07 ± 0.23 ^b^	18.79 ± 0.12 ^d^	11.76 ± 0.71 ^c^	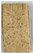
0.8%	61.29 ± 0.25 ^d^	7.39 ± 0.12 ^a^	17.95 ± 0.23 ^e^	14.96 ± 1.21 ^b^	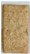
1.0%	59.95 ± 0.14 ^e^	7.75 ± 0.33 ^a^	17.41 ± 0.68 ^e^	16.25 ± 0.63 ^a^	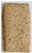

Note: Different lowercase letters indicate significant differences.

**Table 3:** Effects of GSE addition levels on the thickness, diameter, and spread factor of the biscuit.

GSE Addition Levels	Diameter (mm)	Thickness (mm)	Spread Factor
0	135.0 ± 1.2 ^b^	18.0 ± 0.6 ^a^	7.5 ± 0.89 ^d^
0.2%	138.0 ± 5.2 ^ab^	15.0 ± 0.4 ^b^	8.6 ± 0.42 ^c^
0.4%	140.0 ± 4.8 ^a^	15.0 ± 1.5 ^b^	9.3 ± 0.59 ^b^
0.6%	141.0 ± 2.5 ^a^	13.0 ± 1.6 ^c^	10.8 ± 0.53 ^b^
0.8%	142.0 ± 1.8 ^a^	12.0 ± 0.8 ^d^	11.8 ± 0.35 ^a^
1%	143.0 ± 4.6 ^a^	12.0 ± 1.5 ^d^	11.9 ± 1.1 ^a^

Note: Different lowercase letters indicate significant differences.

## Data Availability

The original contributions presented in this study are included in the article. Further inquiries can be directed to the corresponding author.
